# Supplementary material for: Abscisic Acid, Microtubules and Phospholipase D-Solving a Cellular Bermuda Triangle
Source: Int J Mol Sci. 2024 Dec 31;26(1):278. doi: 10.3390/ijms26010278 (PMC11720312; doi:10.3390/ijms26010278)
Supplement: Supplementary file 1 [file ijms-26-00278-s001.zip › Liu_Supplemental_Figure_S1.pptx]

## Slide 1
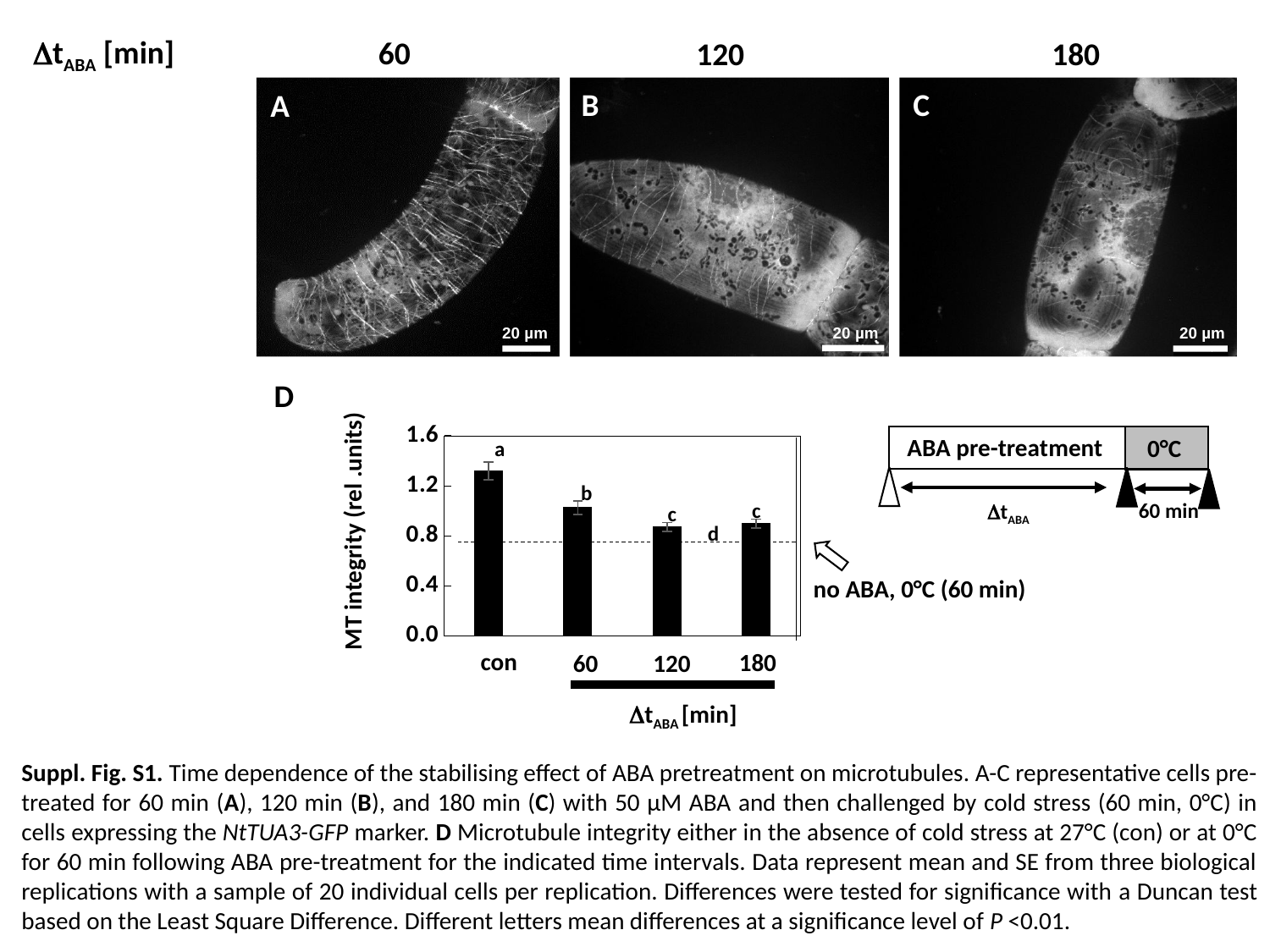

DtABA [min]
60
120
180
B
C
A
50 μ mol ABA 60min+cold 1h
20 µm
20 µm
20 µm
MT integrity (rel .units)
D
### Chart
| Category | |
|---|---|
| CK | 1.3188466413669253 |
| ABA-60min+cold-60min | 1.0249912959277478 |
| ABA-2h+cold-60min | 0.8698 |
| ABA-3h+cold-60min | 0.8988 |ABA pre-treatment
0°C
a
b
c
60 min
DtABA
c
d
no ABA, 0°C (60 min)
con
180
60
120
DtABA [min]
Suppl. Fig. S1. Time dependence of the stabilising effect of ABA pretreatment on microtubules. A-C representative cells pre-treated for 60 min (A), 120 min (B), and 180 min (C) with 50 µM ABA and then challenged by cold stress (60 min, 0°C) in cells expressing the NtTUA3-GFP marker. D Microtubule integrity either in the absence of cold stress at 27°C (con) or at 0°C for 60 min following ABA pre-treatment for the indicated time intervals. Data represent mean and SE from three biological replications with a sample of 20 individual cells per replication. Differences were tested for significance with a Duncan test based on the Least Square Difference. Different letters mean differences at a significance level of P <0.01.
50 μ mol ABA 3h+cold 1h
