# Supplementary material for: Abscisic Acid, Microtubules and Phospholipase D-Solving a Cellular Bermuda Triangle
Source: Int J Mol Sci. 2024 Dec 31;26(1):278. doi: 10.3390/ijms26010278 (PMC11720312; doi:10.3390/ijms26010278)
Supplement: Supplementary file 1 [file ijms-26-00278-s001.zip › Liu_Supplemental_Figure_S2.pptx]

## Slide 1
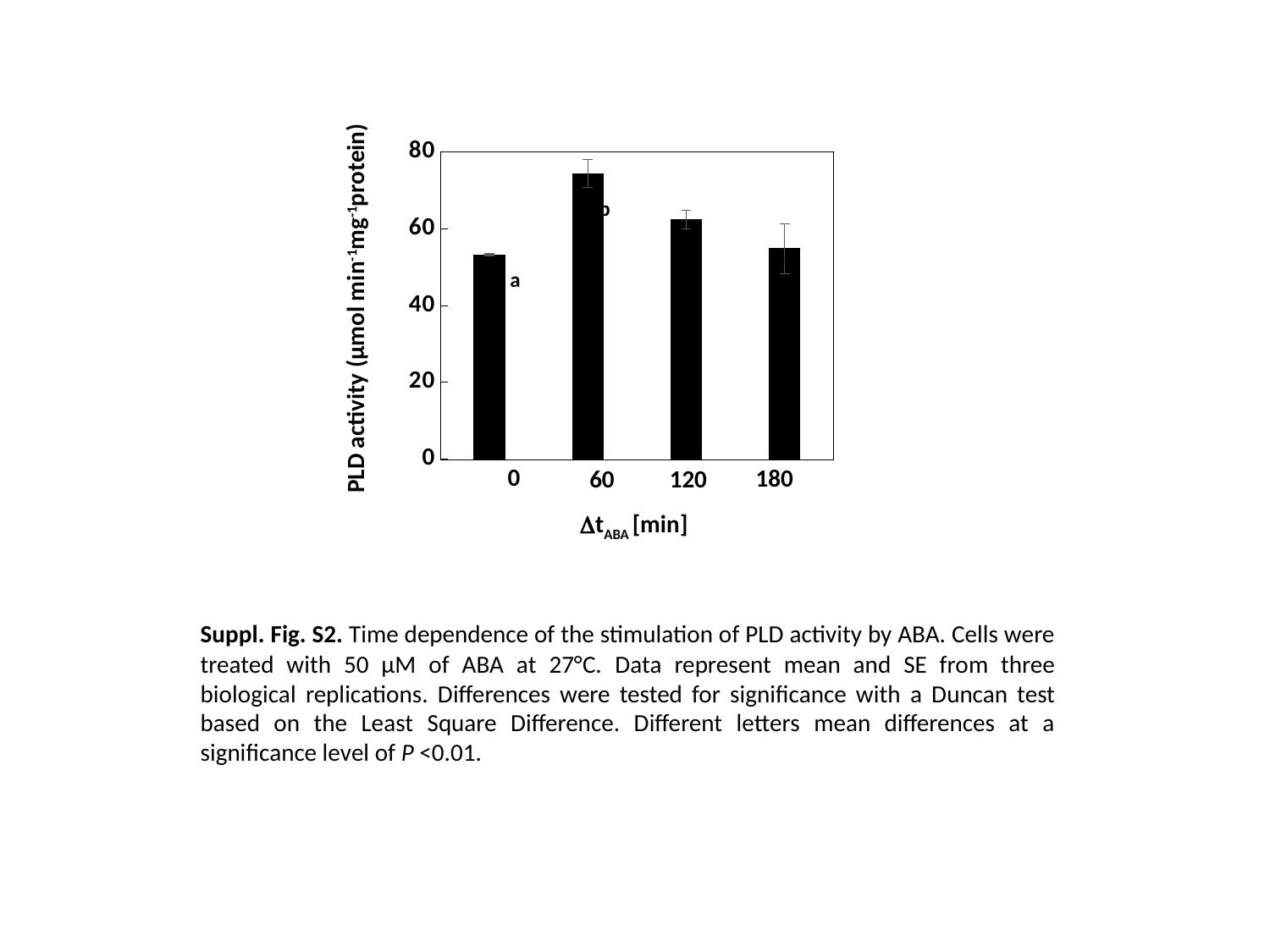

PLD activity (μmol min-1mg-1protein)
### Chart
| Category | |
|---|---|
| CK | 53.18598168060533 |
| ABA-60min | 74.29840810525431 |
| ABA-2h | 62.3257666268419 |
| ABA-3h | 54.75906013540422 |b
ab
a
a
0
180
60
120
DtABA [min]
Suppl. Fig. S2. Time dependence of the stimulation of PLD activity by ABA. Cells were treated with 50 µM of ABA at 27°C. Data represent mean and SE from three biological replications. Differences were tested for significance with a Duncan test based on the Least Square Difference. Different letters mean differences at a significance level of P <0.01.
