# Supplementary figures and images for: Abscisic Acid, Microtubules and Phospholipase D-Solving a Cellular Bermuda Triangle
Source: Int J Mol Sci. 2024 Dec 31;26(1):278. doi: 10.3390/ijms26010278 (PMC11720312; doi:10.3390/ijms26010278)

## Slide 1
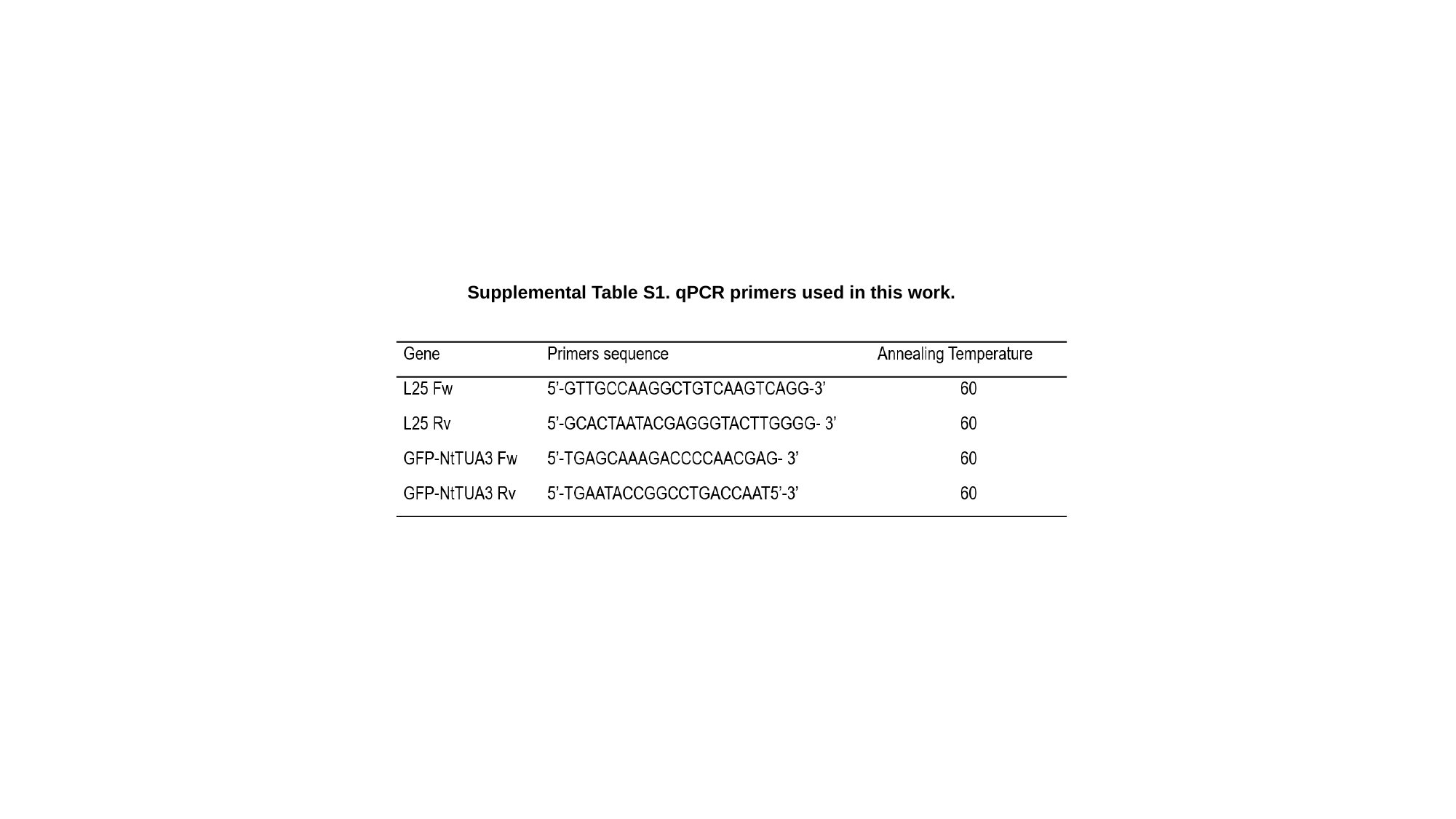

Supplemental Table S1. qPCR primers used in this work.

Supplement: Supplementary file 1 [file ijms-26-00278-s001.zip › Liu_Supplemental_Table_1.pptx]
